# Single-walled carbon nanotube supported Pt-Ru bimetallic superb nanocatalyst for the hydrogen generation from the methanolysis of methylamine-borane at mild conditions

**DOI:** 10.1038/s41598-019-52182-w

**Published:** 2019-10-31

**Authors:** Eda Gokirmak Sogut, Hilal Acidereli, Esra Kuyuldar, Yasar Karatas, Mehmet Gulcan, Fatih Sen

**Affiliations:** 1 Chemistry Department, Faculty of Science, Van Yüzüncü Yıl University, Zeve Campus, 65080 Van, Turkey; 2 Sen Research Group, Biochemistry Department, Faculty of Arts and Science, Dumlupınar University, Evliya Çelebi Campus, 43100 Kütahya, Turkey

**Keywords:** Catalyst synthesis, Heterogeneous catalysis

## Abstract

Several metal nanoparticle based catalysts have been synthesized for catalyzing the hydrogen production process by hydrolysis of methylamine-borane (MeAB). However, there was only one study that catalyzes the producing of hydrogen*via* the methanolysis of MeAB, and it was carried out by our research group. For this reason, in this work, a new catalyst system entitled by single-walled carbon nanotube (SWCNT) supported bimetallic platinum-ruthenium nanoparticles were developed and called as PtRu@SWCNT. These NPs were characterized by several techniques (XRD, XPS, Raman, and TEM), and they were performed for the methanolysis of MeAB with high catalytic activity. The prepared PtRu@SWCNT NPs were also tested in the methanolysis of MeAB at different parameters including different temperatures, catalyst and substrate concentrations, and reusability performance. Experimental results revealed that the new PtRu@SWCNT NPs had excellent catalytic activity and reusability for removing of hydrogen from the methanolysis of MeAB at ambient conditions. According to the obtained data, the turnover frequency is 136.25 mole H_2_/mole PtRu × min, and the activation energy (Ea) is 17.29 kJ/mole. More than 99% of conversion was observed at room temperature.

## Introduction

As a renewable energy source, hydrogen promises to be a carrier of energy for the future^1,2^. However, since hydrogen is light and has a secure storage problem, there are some disadvantages in the application phase^3–5^. For this reason, intensive studies are being carried out for suitable chemicals with high gravimetric hydrogen density for portable and stationary applications^6^. Recently, many chemical hybrid solid hydrogen storage substances such as ammonia-borane (AB), dimethylamine borane (DMAB), methylamine-borane (MeAB) with B-N additives were investigated the situated application^7–11^. The reason for the investigation of these structures is due to the high hydrogen content of the protic N-H, hybrid B-H structures in multiple structures^12–24^. The simplest B-N compound is AB, which has a hydrogen mass of 19.6% and low molecular weight (30.9 g/Mol)^25,26^. They have a stable structure under ambient conditions with metal amido-borane, MeAB, and dimethylamine-borane. MeAB (CH_3_NH_2_-BH_3_) is an AB derivative having 11.1% hydrogen mass and stable to operating conditions^27,28^. Solvent (methanolysis and hydrolysis) and solid phase thermolysis reactions were applied from MeAB in the hydrogen production^29,30^. In the presence of the suitable catalyst, the hydrogen release in the solvolysis of MeAB yields 3 moles of hydrogen for 1 mole of MeAB according to following Eqs (1) and (2).

1 $${{\rm{CH}}}_{3}{{\rm{NH}}}_{2}-{{\rm{BH}}}_{3}({\rm{aq}})+2{{\rm{H}}}_{2}{\rm{O}}\to ({{\rm{CH}}}_{3}{{\rm{NH}}}_{3}){{\rm{BO}}}_{2}({\rm{aq}})+3{{\rm{H}}}_{2}({\rm{g}})$$

2 $${{\rm{CH}}}_{3}{{\rm{NH}}}_{2}-{{\rm{BH}}}_{3}+4{{\rm{CH}}}_{3}{\rm{OH}}\to ({{\rm{CH}}}_{3}{{\rm{NH}}}_{3}){\rm{B}}{({{\rm{OCH}}}_{3})}_{4}+3{{\rm{H}}}_{2}({\rm{g}})$$

In the literature, there are several metal nanoparticles based catalysts for catalyzing the hydrogen production process by hydrolysis of MeAB. However, until the present study, there was only one study that catalyzes the producing of hydrogen via the methanolysis of MeAB, and it was carried out by our research group^1^.

In this study, Pt-Ru alloy nanoparticle decorated on SWCNT was synthesized and characterized by several techniques. The prepared new PtRu@SWCNT NPs nanocatalyst was tested effectively to complete dehydrogenation of MeAB by the methanolysis reaction. The methanolysis reaction with the use of the PtRu@SWCNT NPs began without any observing induction time at room conditions. The detailed kinetic study of synthesized nanoparticles for the methanolysis reaction of MeAB catalyzed by PtRu@SWCNT NPs were performed with the help of Arrhenius and Eyring equations.

## Results and Discussion

### The chemical and morphological structure of PtRu@SWCNT NPs

**Figure 1:**
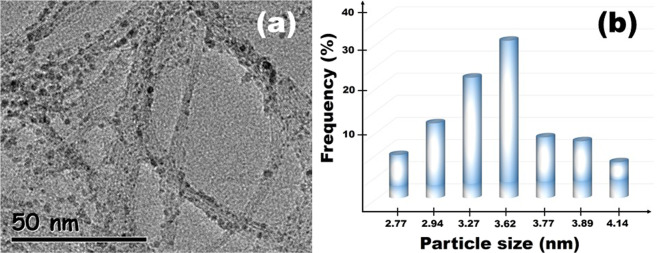
(**a**) TEM pattern and (**b**) Particle size of PtRu@SWCNT NPs nanocatalyst.

In order to reveal the chemical and morphological structure of PtRu@SWCNT NPs, various advanced analytical analysis techniques were conducted, and the details of characterization studies were given in supporting information. Figure 1 shows TEM analysis for PtRu@SWCNT NPs to reveal the mean particle size and distribution of PtRu alloy nanometals on SWCNT. As seen in Fig. 1, the mean particle size of PtRu@SWCNT NPs were found to be 3.62 ± 0.5 nm and this figure also show monodisperse and homogeneous distribution of the Pt and Ru metals on the supporting material. There was no agglomeration of PtRu nanoparticles on SWCNT.

**Figure 2:**
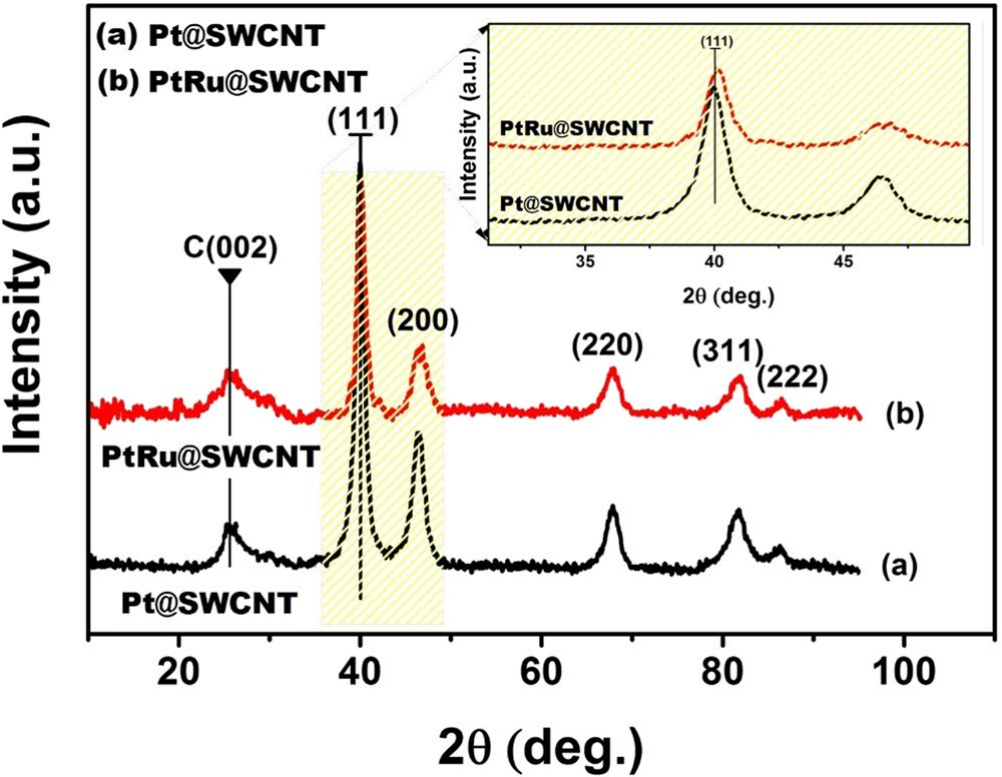
XRD analysis of Pt@SWCNT and PtRu@SWCNT nanocatalysts.

XRD analysis was used to determine the crystalline structure of the monodisperse PtRu@SWCNT NPs nanocatalyst (containing 3.34 ± 0.02 wt % PtRu as founded using ICP-OES). Figure 2 shows XRD patterns of Pt@SWCNT NPs and PtRu@SWCNT NPs. As seen in Fig. 2, the similar XRD patterns were determined for Pt@SWCNT NPs and PtRu@SWCNT NPs, however, there was a small shift to the higher 2θ values which shows the alloy formation of PtRu@SWCNT compared to the Pt@SWCNT after 2^nd^ metal addition. Both of Pt@SWCNT and PtRu@SWCNT have showed face centered cubic (*fcc*) structure and the XRD analysis also revealed the crystalline structures of PtRu@SWCNT NPs after the stabilization of metal ions to metallic forms^31^.

**Figure 3:**
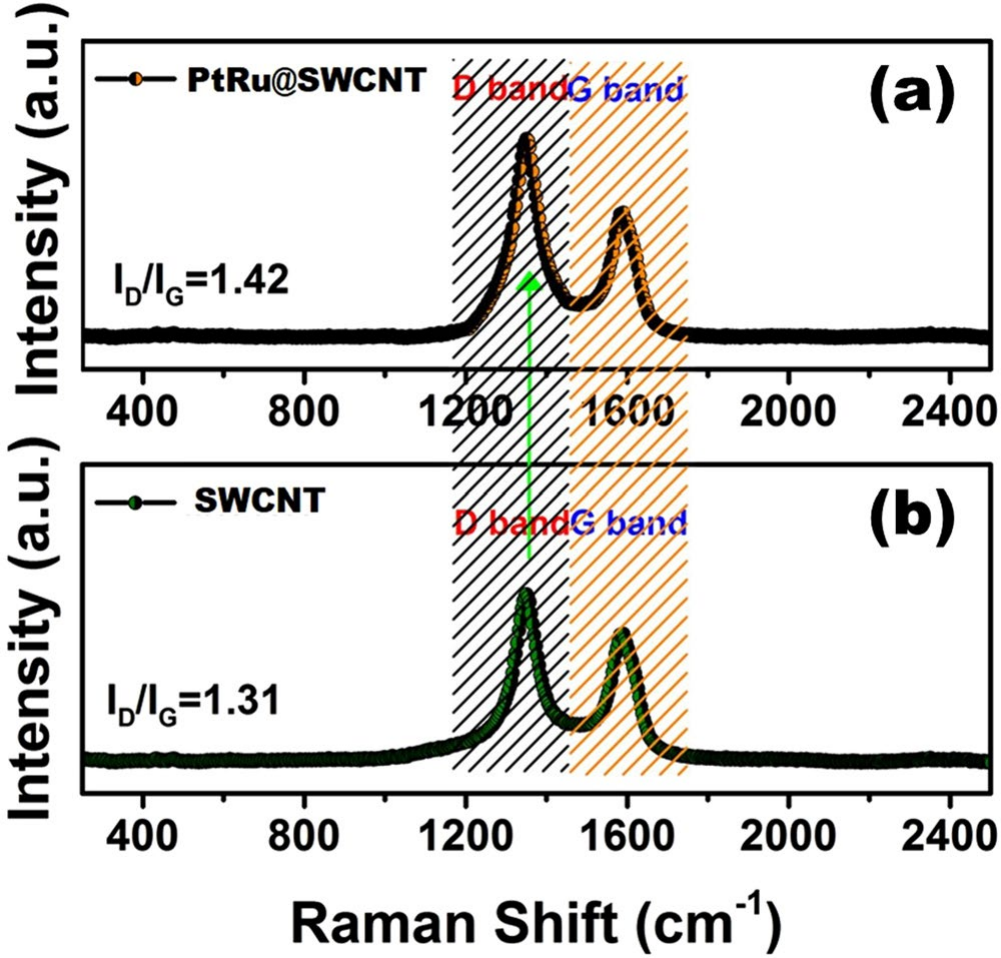
(**a**) Raman spectra of PtRu@SWCNT NPs nanocatalyst and (**b**) SWCNT support material.

The Raman spectroscopy was shown in Fig. 3. The peaks observed at 1349 and 1589 cm^−1^, correspond to the D and G bands of carbon based materials, respectively. The intensity of graphite and the degree of graphitization of the carbonaceous materials represent the density ratio of the D-G band (I_D_/I_G_). After the functionalization of SWCNT with PtRu nanoparticles, I_D_/I_G_ value increased from 1.31 to 1.42. The change in this ratio means the increase in deficiency of SWCNT which supports their functionalization with nanoparticles^32^.

**Figure 4:**
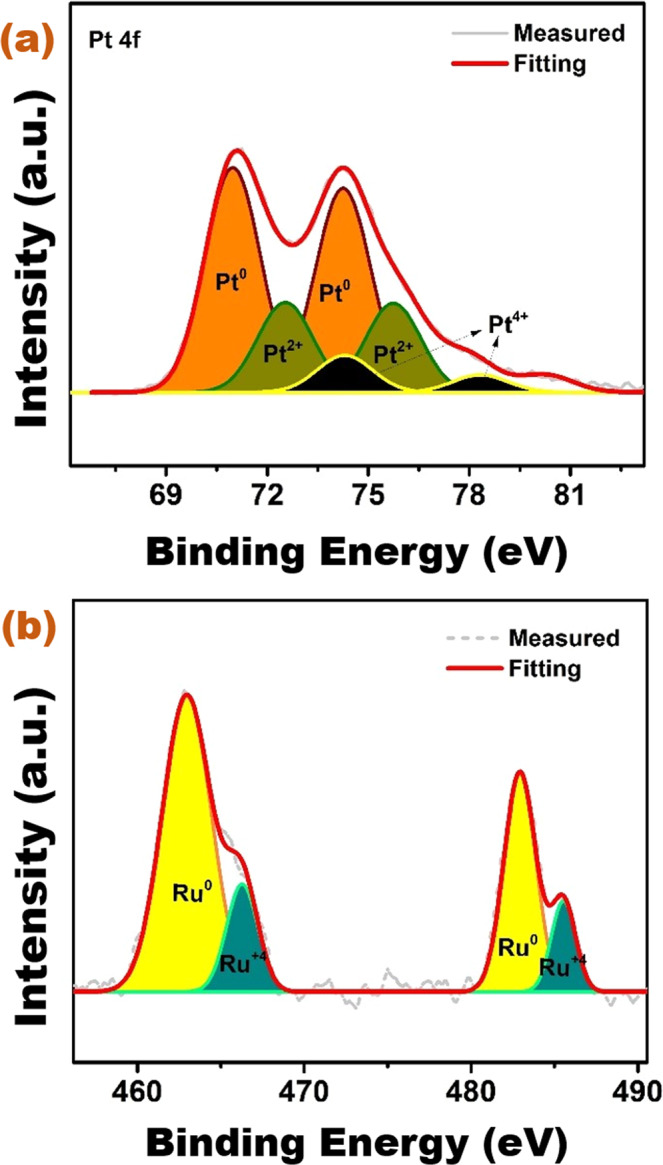
(**a**) Pt 4 f and (**b**) Ru 3p region XPS spectra of PtRu@SWCNT NPs nanocatalyst.

For further investigations about the oxidation state of metals in PtRu@SWCNT NPs nanocatalyst, X-ray photoelectron spectroscopy (XPS) analyses were conducted. The electronic features of Pt and Ru, and their synergistic effect with SWCNT support material were investigated using XPS analysis. Figure 4 shows the XPS spectrum of PtRu@SWCNT NPs nanocatalyst. The oxidation state analysis of Pt and Ru in the PtRu@SWCNT NPs superb nanocatalyst was analyzed with Pt 4 f and Ru 3p regions in the spectrum. Pt 4 f and Ru 3p regions at XPS spectrum of the PtRu@SWCNT NPs give three doublets at 71.0 (metallic), 72.4 (Pt^2+^) and 73.9 eV (Pt^4+^) and two doublets at about 464.4 (metallic) − 467.5 eV (Ru^4+^), respectively^31,32^.

### The methanolysis of MeAB catalyzed by PtRu@SWCNT NPs nanocatalyst

**Figure 5:**
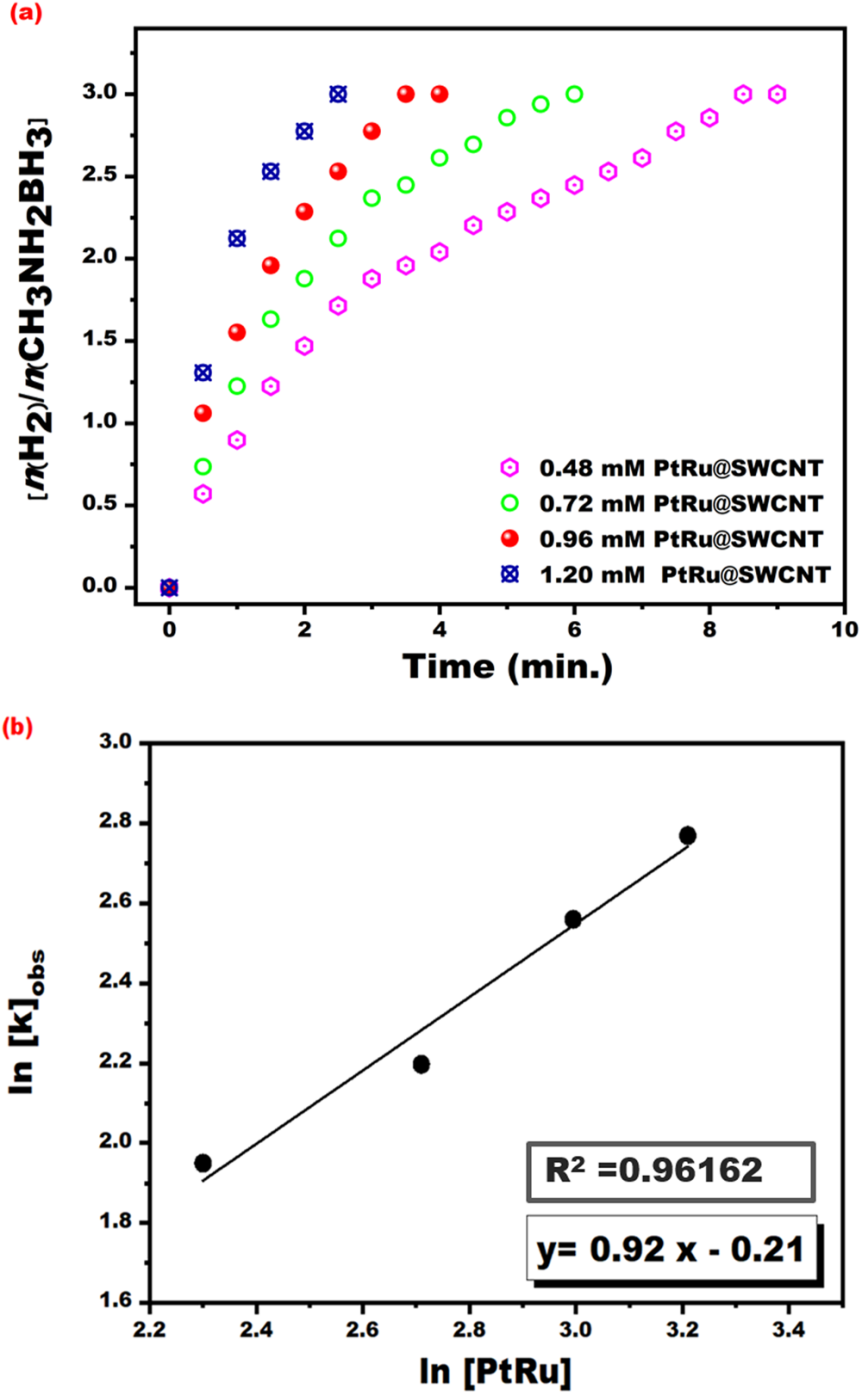
(**a**) The plot for hydrogen evolve from MeAB dehydrogenation reaction (50 mM in 5 mL dry methanol) and (**b**) plot of lnk_*obs*_ versus ln[PtRu] for the methanolysis of MeAB with different PtRu@SWCNT NPs concentrations ([PtRu@SWCNT] = 0.48, 0.72, 0.96 and 1.2 mM) at room temperature.

For the catalytic performance experiments, PtRu@SWCNT NPs nanocatalyst (0.96 mM) was added to a vacuum Schlenk tube. 4 mL of pre-dried methanol added to Schlenk tube and closed with the septum. 50 mM MeAB (0.25 mmol, 11.25 mg) was dissolved in 1 mL of dry methanol. In the presence of dissolved MeAB and N_2_ gas, it is placed in a jacketed Schlenk. Then the timer is started at t = 0. The released hydrogen gas amount was recorded using a cylinder burette. The experimental results obtained from different PtRu@SWCNT NPs nanocatalyst concentrations (0.48–1.20 mM) in the methanolysis of MeAB at mild conditions were given in Fig. 5. The hydrogen evolves began no observing any induction time as seen in Fig. 5(a). The complete hydrogen releasing from MeAB catalyzed by PtRu@SWCNT NPs nanocatalyst occurred in a very little time like 3.5 min at mild conditions. The plot obtained from the experiments carried out at different PtRu@SWCNT NPs nanocatalyst concentrations is given in Fig. 5(b) (lnk_*obs*_ versus to ln[PtRu]) and the obtained plot is linear. The slope of the plot was found to be 0.92. According to experimental results, the rate of MeAB methanolysis, in the presence of PtRu@SWCNT NPs nanocatalyst was determined as 0.92^nd^ depending on the concentration of PtRu@SWCNT NPs superb nanocatalyst.

**Figure 6:**
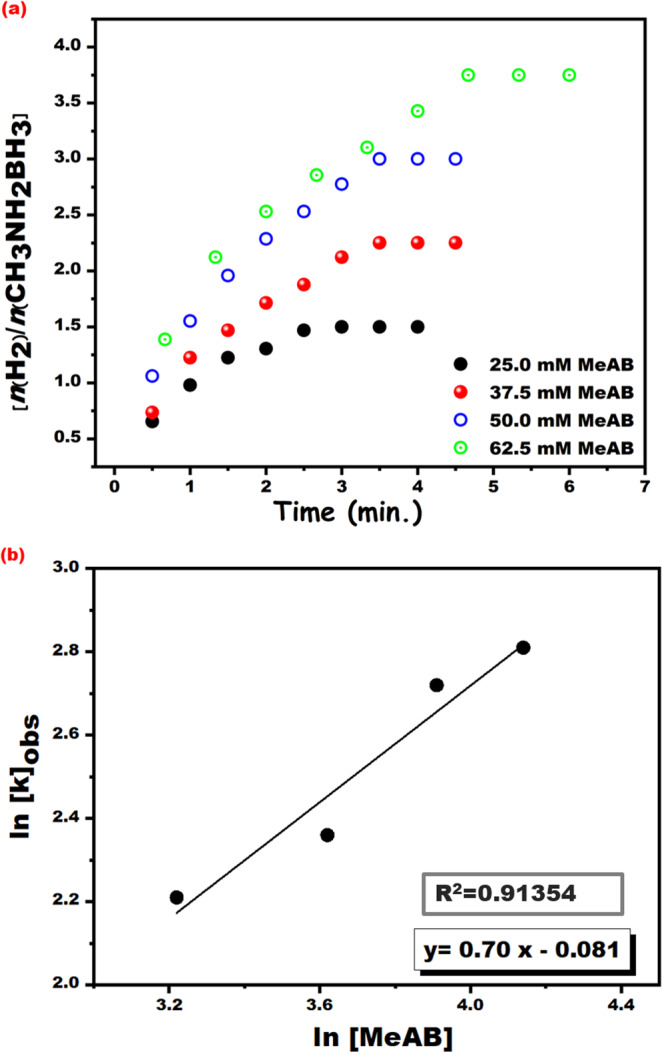
(**a**) The plot for hydrogen evolve from the methanolysis of MeAB (50 mM) catalyzed by PtRu@SWCNT NPs ([PtRu@SWCNT] = 0.96 mM in 5 mL dry methanol) and (**b**) the graph of lnk_*obs*_ versus ln[MeAB] for the methanolysis of MeAB performed with various substrate concentrations ([MeAB] = 25.0, 37.5, 50.0 and 62.5 mM) at room temperature.

Figure 6(a) indicates the volume of generated hydrogen versus time for the methanolysis of MeAB catalyzed by PtRu@SWCNT NPs nanocatalyst, started with different MeAB concentrations (25.0, 37.5, 50.0 and 62.5 mM) in dry methanol at ambient conditions. A linear graph of lnk_*obs*_ versus ln [MeAB] plot was acquired from the Fig. 6(b), and a 0.70 of slope was obtained from Fig. 6. The results demonstrated that the rate of methanolysis of MeAB catalyzed by PtRu@SWCNT NPs nanocatalyst was suitable to the 0.70^th^ order equation, depending on the concentration of MeAB. Based on the results mentioned above, catalytic rate law for hydrolysis of MeAB with PtRu@SWCNT NPs nanocatalyst was obtained as follows:

$$-{\rm{d}}[{{\rm{CH}}}_{3}{{\rm{NH}}}_{2}-{{\rm{BH}}}_{3}]/{\rm{dt}}=+{\rm{d}}[{{\rm{H}}}_{2}]/3{\rm{dt}}={{\rm{k}}}_{obs}{[{\rm{PtRu}}@{\rm{SWCNT}}{\rm{NPs}}]}^{0.92}{[{\rm{MeAB}}]}^{0.70}$$

**Figure 7:**
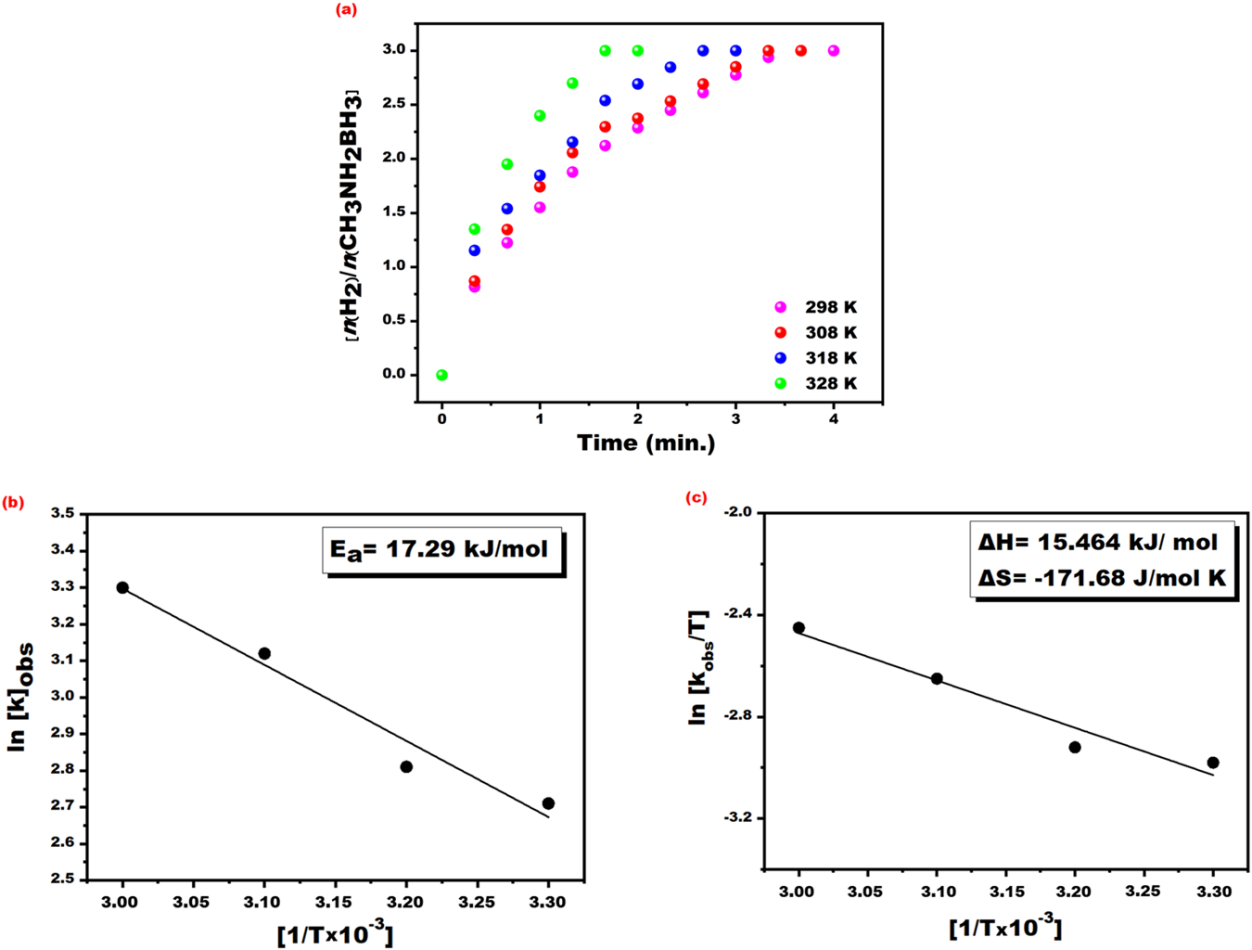
(**a**) The plot for hydrogen evolve from MeAB dehydrogenation reaction (50 mM in 5 mL dry methanol), PtRu@SWCNT NPs ([PtRu@SWCNT] = 0.96 mM in 5 mL dry methanol) and performed at different temperatures of 298, 308, 318 and 328 K, (**b**) Arrhenius and (**c**) Eyring plot for the methanolysis reaction of MeAB.

**Figure 8:**
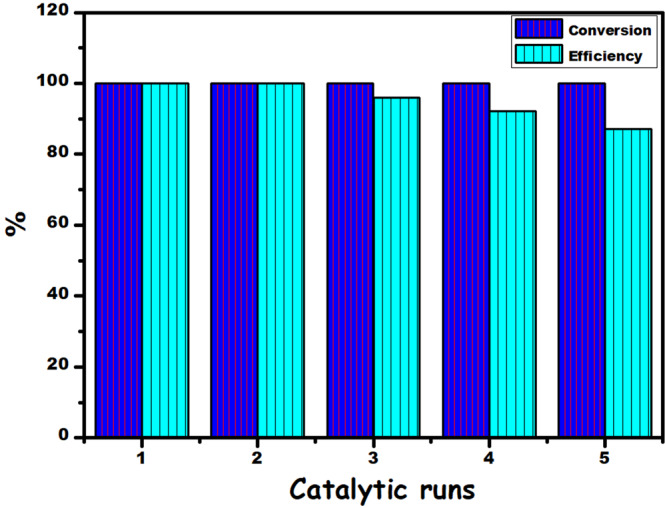
The performance of recyclability and conversion % of PtRu@SWCNT NPs nanocatalyst for the methanolysis reaction of MeAB.

To set the optimal temperature for the methanolysis of MeAB catalyzed with PtRu@SWCNT NPs various experiments were carried out containing 50 mM MeAB and 0.96 mM PtRu@SWCNT NPs nanocatalyst at different temperatures (25–55 °C). The results obtained from the experiment conducted at different temperatures and Arrhenius – Eyring equations were used to calculate activation (activation energy (Ea) enthalpy (ΔH^#^) and entropy (ΔS^≠^)) and the kinetic parameters of MeAB catalyzed with PtRu@SWCNT NPs nanocatalyst. The results of the experiments conducted at different temperatures are given in Fig. 7(a). As seen in this figure, when increased temperatures, the catalytic activity of PtRu@SWCNT NPs nanocatalyst were increased. The activation energy (Ea) for the methanolysis of MeAB catalyzed with PtRu@SWCNT NPs nanocatalyst was calculated to be 17.29 kJ/mole using Arrhenius plot (given in Fig. 7(b)). Additionally, the observed reaction constant given in Fig. 7(c) was used to calculate enthalpy and entropy values for the methanolysis of MeAB catalyzed with PtRu@SWCNT NPs nanocatalyst, and these values were found to be ΔH^#^ = 15.46 kJ/mole and ΔS^#^ = −171.68 J/(mole × K), respectively. To test the stability and recyclability of PtRu@SWCNT NPs nanocatalyst in the methanolysis of MeAB at room temperatures, the same concentration of MeAB was subsequently added the completed experiment reaction after the previous run. Finally, the recyclability performance of PtRu@SWCNT NPs nanocatalyst has been shown to maintain its initial activity (87%) and provides high conversion (>99%) at the end of the 5th catalytic cycle (Fig. 8).

The initial turn-over frequency (TOF_*initial*_) for PtRu@SWCNT NPs was found to be 8175 h^−1^ (136.25 min^−1^) at room temperatures and the calculated this TOF value were compared the TOF values present in literature as seen in Table 1. This TOF value is higher than the other study used for the methanolysis of MeAB at room conditions. As a result, the synthesized PtRu@SWCNT NPs nanocatalyst exhibited a superior catalytic activity compared the previous catalyst used for the methanolysis of MeAB. This unique catalytic activity can be ascribed to the large surface area of the catalysts, the synergic effects of alloy metals (Pt-Ru) with SWCNT and ultrafine structure.

**Table 1:** The catalysts tested for their catalytic activity and initial TOF values in the dehydrogenation of DMAB, hydrolysis and methanolysis of MeAB at room temperatures.

Catalyst	TOF*	Ea**	Reaction Type	Ref.
RhCl_3_	7.9	ND	dehydrogenation of DMAB	^7^
Pd/C	2.8	ND	dehydrogenation of DMAB	^7^
Trans-RuMe_2_(PMe_3_)_4_	12.4	ND	dehydrogenation of DMAB	^7^
IrCl_3_	0.3	ND	dehydrogenation of DMAB	^7^
Cp_2_Ti	12.3	ND	dehydrogenation of DMAB	^8^
RhCl(PPh_3_)_3_	4.3	ND	dehydrogenation of DMAB	^7^
RuCl_3_.3H_2_O	2.7	ND	dehydrogenation of DMAB	^9^
Pt@PANI-rGO	42.94	ND	dehydrogenation of DMAB	^10^
Pt@AC	28.93	ND	dehydrogenation of DMAB	^11^
Pt@VC	23.14	ND	dehydrogenation of DMAB	^11^
Rh/graphene	146	16.4	hydrolysis of MeAB	^24^
Ru/MCM-41	47.60	ND	hydrolysis of MeAB	^34^
Rh_1_Ni_7.5_/graphene NPs	ND	31.26	hydrolysis of MeAB	^35^
Cu/*nano*-MIL-101	4.3	34.1	hydrolysis of MeAB	^23^
Cu_0.1_@Co_0.45_Ni_0.45_/graphene NPs	ND	50.75	hydrolysis of MeAB	^36^
Co_0.9_Ni_0.1_/graphene NPs	ND	26.78	hydrolysis of MeAB	^14^
Cu_0.2_@Co_0.8_/rGO	ND	39.69	hydrolysis of MeAB	^37^
Ag@CoNiFe/graphene	ND	33.53	hydrolysis of MeAB	^38^
Cu_12.6_@Fe_9.8_Co_38.8_Ni_38.8_/graphene	ND	39.69	hydrolysis of MeAB	^39^
Rh/*nano*-ZrO_2_	17.52	51.45	methanolysis of MeAB	^1^
**PtRu@SWCNT NPs**	**136.25**	**17.29**	methanolysis of MeAB	**This study**

*Turnover frequency (mole of H_2_/(mole of catalyst × min)), **Activation energy (kJ/mole).

## Conclusions

In summary, even though several metal nanoparticles based catalysts have been synthesized for catalyzing the hydrogen production process by hydrolysis of MeAB, there was only one study related to the methanolysis of MeAB. For above reason, the superb PtRu@SWCNT NPs nanocatalyst was synthesized and tested as an effective catalyst in the methanolysis of MeAB with an easy and facile technique at mild conditions. With this report, a new and effective PtRu@SWCNT NPs nanocatalyst was developed for the methanolysis reaction of MeAB with complete hydrogen evolve at mild conditions. PtRu@SWCNT NPs superb nanocatalyst showed very high catalytic activity in the dehydrogenation of MeAB in dry methanol environment. The rate law of catalytic methanolysis of MeAB including PtRu@SWCNT NPs superb nanocatalyst was obtained as -d[CH_3_NH_2_BH_3_]/dt = +d[H_2_]/3dt = k_*obs*_ [PtRu@SWCNT NPs]^0.92^ [MeAB]^0.70^. The activation energy, enthalpy, and entropy of the methanolysis of MeAB were found to be 17.29 kJ/mole, 15.46 kJ/mole, and −171.68 J/(mole × K), respectively. The initial TOF value of superb PtRu@SWCNT NPs nanocatalyst was found to be 8175 h^−1^ (136.25 min^−1^) as a record catalytic activity for methanolysis of MeAB, at 298 K in literature as shown in Table 1. This unique catalytic activity can be ascribed to the large surface area of the catalysts, the synergic effects of alloy metals (PtRu) with SWCNT and ultrafine structure. This new, effective and superb PtRu@SWCNT NPs nanocatalyst can be used for evolving hydrogen from MeAB as a solid hydrogen source in fuel cell applications at mild conditions. The catalysts exhibiting high catalytic activity have significant importance in the hydrogen technologies.

## Experimental

### Preparation of methylamine-borane (MeAB, CH_3_NH_2_-BH_3_)

Yang *et al*. reported a method for the synthesis of MeAB^33^. For this aim, 0.1 mole (3.88 g) of NaBH_4_ was weighed into a 250 mL two-necked flask, and 200 mL of anhydrous tetrahydrofurane (THF) was added. After stirring for 30 minutes at 25 °C, 0.1 mol (6.752 g) of methylamine hydrochloride was added through 24 hours at 25 °C under N_2_ atmosphere. The mixture was filtered, and the liquid phase was evaporated, allowing the THF to leave the medium. THF was entirely removed from the medium, and 100 mL of dry ether was added. The mixture was stirred for 2 hours in a cryostat system at a temperature of 0 °C. At the end of this period, the solid phase was re-filtered. The supernatant was left at room temperature for complete removal of water. When the supernatant was evaporated entirely, the white solids were formed as shown in following scheme (3).

3 $${{\rm{CH}}}_{3}{{\rm{NH}}}_{2}\cdot {\rm{HCl}}+{{\rm{NaBH}}}_{4}\to {{\rm{CH}}}_{3}{{\rm{NH}}}_{2}-{{\rm{BH}}}_{3}+{{\rm{H}}}_{2}({\rm{g}})+{\rm{NaCl}}$$

### Preparation of single-walled carbon nanotube supported platinum-ruthenium nanoparticles (PtRu@SWCNT NPs)

In the preparation of the new PtRu@SWCNT NPs nanocatalyst, an easy and facile one-step reduction technique was used at room conditions. Briefly, a solution containing 30 mg K_2_PtCl_4_, 30 mg RuCl_3_∙*x*H_2_O_,_ and 60 mg SWCNT were mixed in 20 mL of water. After that, a solution containing NaBH_4_ was added to the mixture and waited until the bubble formation was finished. After that, black colored PtRu@SWCNT NPs superb nanocatalyst was obtained. The resulting mixture was filtered, and the obtained solid residue was washed with plenty of deionized water (3 × 10 mL), dried at inert medium at 80 °C.

## Supplementary information

Single-walled carbon nanotube supported Pt-Ru bimetallic superb nanocatalyst for the hydrogen generation from the methanolysis of methylamine-borane at mild conditions
