# Supplementary material for: Single-walled carbon nanotube supported Pt-Ru bimetallic superb nanocatalyst for the hydrogen generation from the methanolysis of methylamine-borane at mild conditions
Source: Sci Rep. 2019 Oct 31;9:15724. doi: 10.1038/s41598-019-52182-w (PMC6823424; doi:10.1038/s41598-019-52182-w)
Supplement: Supplementary file 1 — Single-walled carbon nanotube supported Pt-Ru bimetallic superb nanocatalyst for the hydrogen generation from the methanolysis of methylamine-borane at mild conditions [file 41598_2019_52182_MOESM1_ESM.docx]

Electronic Supplementary Material

**Single-walled carbon nanotube supported Pt-Ru bimetallic superb nanocatalyst for the hydrogen generation from the methanolysis of methylamine-borane at mild conditions**

Eda Gokirmak Sogut^a^, Hilal Acidereli^b^, Esra Kuyuldar^b^, Yasar Karatas^a^, Mehmet Gulcan^a^^^[[1]](#footnote-1)^^, Fatih Sen^b*^

*^a^Chemistry Department, Faculty of Science, Van Yüzüncü Yıl University, Zeve Campus 65080 Van, Turkey;* *^b^Sen Research Group, Biochemistry Department, Faculty of Arts and Science, Dumlupınar University, Evliya Çelebi Campus, 43100 Kütahya, Turkey*

**Materials and Methods**

Potassium hexachloroplatinate (IV) (K_2_PtCl_6_, 98%), ruthenium (III) chloride hydrate (RuCl_3_·*x*H_2_O), sodium borohydride (NaBH_4_, 98%), single-walled carbon nanotube (SWCNT), tetrahydrofuran and methylamine hydrochloride were purchased from Sigma-Aldrich. Tetrahydrofuran (THF) was distilled with sodium benzophenone in an Ar atmosphere, then stored in a dry box (H_2_O, O_2_<1 ppm). All solutions were prepared with distilled water, and distilled water was used in whole experiments.

The TEM images have been obtained by a JEOL 200 kV TEM instrument. Powder X-ray Diffraction (P-XRD) analysis was performed using a diffractometer with Ultima + theta + theta high resolution goniometer, Cu Kα radiation (Bruker AXS D8-Advanced, 45 kV, 40 mA, λ= 1.54056Å). In the characterization studies, a solution was prepared as described in the above section 2.2 for examination; this solution was centrifuged at 8000 rpm for 15 minutes. The nanoparticles obtained from the centrifuged mixture are cleaned using purified water and ethanol to remove contaminants and the excess of the nanoparticle of PtRu@SWCNT. The obtained nanoparticles were re-dissolved in 5 mL of pure water. One drop of the resulting mixture was added dropwise onto a copper grid and evaporated to dryness under nitrogen. TEM images were used to calculate the mean particle size of PtRu@SWCNT NPs nanocatalyst, to do this, the particles in the TEM images were calculated by counting them. The absorption experiments of the platinum (0) and ruthenium (0) nanoparticles stabilized with SWCNT were performed with a double-beam Perkin Elmer Lambda UV-VIS-NIR spectrophotometer. FT-IR analyses were taken by Perkin Elmer Spectrum 2. X-ray Photoelectron Spectroscopy (XPS) analysis was done using physical electronics 5800 spectrometers consisted of the hemispherical analyzer and having monochromatic Al Kα radiation (1486.6 eV, the X-ray tube working at 15 kV, 350 W and pass energy of 23.5 keV). Nuclear magnetic resonance (NMR) analysis was performed by a Bruker Avance DPX 400 MHz spectrometer (400.1 MHz for ^1^H NMR; 100.6 MHz and 128.2 MHz for ^11^B NMR). BF_3_.(C_2_H_5_)_2_O and Si(CH_3_)_4_ were taken to use as an internal reference for ^11^B-NMR and ^1^H-NMR chemical shifts.

1. Corresponding authors: [mehmetgulcan65@gmail.com](mailto:mehmetgulcan65@gmail.com) (M. Gülcan), [fatihsen1980@gmail.com](mailto:fatihsen1980@gmail.com) (F. Şen) [↑](#footnote-ref-1)
